# Dr. G. H. Kilburne

**Published:** 1855-07

**Authors:** 


We learn from a Vermont paper, with deep regret, that Dr. G. H. Kilburne, dentist of Montpelier, died the 9th of April, in the 32d year of his age, leaving a wife, one child, and other relatives and friends to mourn his early loss. The disease which terminated his mortal being was pulmonary consumption. Dr. K., we believe, was a highly esteemed member of his profession, but the writer of this was not sufficiently acquainted with him to enable him to speak of his merits as he would be glad to do.

